# Socio-behavioral factors linked to willingness to use new male contraceptives: a cross-sectional survey of men and female partners across four low- and middle-income countries

**DOI:** 10.1016/j.contraception.2026.111507

**Published:** 2026-05-22

**Authors:** Brian T. Nguyen, Nancy Kidula, Nathalie Kapp, Khurshed Nosirov, Mohamed Mahmoud Ali, Abdellatif Maamri, Kwasi Adu Manu, Kojo Asamoah Boateng, Marcela Sánchez, Paola C. Montenegro, Koffi E. Dzotsi, James Kiarie

**Affiliations:** 1 Keck School of Medicine of the University of Southern California; California, USA; 2 The UNDP-UNFPA-UNICEF-WHO-World Bank Special Programme of Research, Development and Research Training in Human Reproduction, Department of Sexual and Reproductive Health and Research, WHO, Geneva, Switzerland; 3 International Planned Parenthood Federation, London, UK; 4 Association Marocaine de Planification Familiale/UMPO; 5 Planned Parenthood Association of Ghana; 6 Profamilia, Colombia; 7 Association Togolaise pour le Bien-Etre Familial

**Keywords:** male contraception, men’s health, men’s reproductive health, gender

## Abstract

**Objective:**

Numerous population-level surveys support willingness to use new male contraceptives (MC) among men and their female partners across a range of cultures and geographies, despite unproven efficacy and uncertain risk. Whether such willingness is related to attitudes about gender equity and/or key reproductive experiences warrants further exploration. We explore socio-behavioral factors linked to variations in willingness to use new MCs in Colombia, Ghana, Morocco, and Togo.

**Methods:**

We conducted approximately two thousand surveys examining new MC attitudes with sexually active, reproductive age (18-50 years) men and their female sexual partners, recruited from both clinic and community settings from July-September 2023. We additionally collected socio-demographics, reproductive histories, and contraceptive attitudes and behaviors, examining their associations with willingness to use new MCs via regression modeling.

**Results:**

Nearly 75% (72.3% male, 76.2% female partners; n=1444/1945) of survey respondents reported willingness to use new MCs. Willingness ranged from 69.4% in Togo to 82.2% in Colombia. Factors independently linked to willingness to use new MCs among men included having multiple current sex partners and having been involved in an unintended pregnancy; those for female partners included wanting to avoid pregnancy, and current use of or desire for contraception. For both partners, willingness to use new MCs was linked to increasing gender equitable attitudes.

**Conclusions:**

Regardless of gender, geography, class, culture, and reproductive experience, the significant association of gender equitable attitudes on willingness to use new male contraceptives suggests a potential role for gender equity initiatives to increase their uptake when available.

## 1 Background

Male-controlled contraceptive methods (MCs)—external condoms, withdrawal, and vasectomy—comprise approximately 30% of methods used to prevent pregnancy among women in both the United States and in low- and middle-income countries [1,2]. The proportion of men desiring to share contraceptive responsibility, however, may extend beyond use of MCs to contributing to contraceptive decision-making and supporting their partner’s contraceptive use. Condoms remain costly and carry stigma [3], withdrawal provides limited protection, and vasectomy is permanent and can be difficult to access [4]. Consequently, men’s potential to share contraceptive responsibilities may be limited by the number and characteristics of available MCs, as well as social and gender norms[5].

While hormonal male contraceptives that suppress spermatogenesis (e.g., oral pills, intramuscular injections, and transdermal gels) are closest to market, researchers are exploring contraceptive targets throughout the male reproductive system. The progress and range of research reflect growing interest in new MCs, which are likely to be available within 10-20 years. Trials of investigational, hormonal MCs show high efficacy and tolerability [6,7]. Acceptability surveys of participants also show high satisfaction for various routes of administration—injections [6], topical gels,[8], and pills [9,10]. While the experiences of trial participants and their partners reflect ideal clinical-trial conditions, numerous population-level surveys conducted over the last two decades consistently report strong interest in using new, hypothetical MCs among men [11] [12], and their female partners [13].

In the most definitive examination of demand for new MCs, a multi-national study in 2022, recruited more than 15,000 men from Bangladesh, Vietnam, Kenya, Nigeria, DR Congo, Côte d’Ivoire, and the United States, noting interest in trying new MCs within the first year of availability among 61% of respondents [14]. Notably, this survey used discrete choice experiments to examine the influence of product characteristics on willingness to use new MCs, with the most important factor being administrative route. Various potential side effects (e.g., energy, weight, sex drive, mood swings) contributed the least to men’s preferences, challenging beliefs about men’s fear of or unwillingness to experience side effects. Willingness to use new MCs reached as high as 76% in Nigeria and Bangladesh—countries with lower levels of gender equality [15]—in contrast to the United States with its middle range gender equality level and lowest level of willingness at 49%. As gender disparities might influence men’s involvement in family planning, these findings are an opportunity to further explore socio-behavioral factors linked to willingness to use new MCs among potential users in low- and middle-income countries.

## 2 Materials and Methods

### 2.1 Objectives

We conducted a multi-national survey in low and middle-income countries with the objective of: (1) examining men and their female partner’s willingness to use new MCs, and (2) exploring the influence of sociodemographic characteristics, reproductive factors, and gender norms on willingness to use new MCs.

### 2.2 Survey Method

We partnered with the International Planned Parenthood Federation’s (IPPF) Member Associations in Colombia (Profamilia), Ghana (Planned Parenthood of Ghana), Morocco (Moroccan Association of Family Planning), and Togo (Association Togolaise pour le Bien-Etre Familial). The regions represented by these associations were selected for contrasting levels of gender equity [16] and limited contraceptive use [17], particularly among the Arab-Muslim population of Morocco, thereby providing an important perspective on social and gender-based factors. We anticipated recruiting approximately 250 couples from each country for a total of 1000 male and 1000 female partners. As an exploratory, multi-country study, this analysis was not designed to generate nationally representative estimates or to meet formal power thresholds. Sample sizes were determined pragmatically to balance resource constraints with the aim of capturing heterogeneity within each country and adequacy for multivariable logistic regression modeling, with sufficient outcomes to support inclusion of key sociodemographic and reproductive behavioral covariates. Due to budgetary limitations and previous publications of male contraceptive attitudes in Asian countries like China and Hong Kong [18], Asian countries were not included.

The World Health Organization (WHO) and IPPF London Office served as coordinating centers; local investigators were responsible for the field work at each respective study center. The study received approval from WHO’s ethics review committee and the institutional review boards affiliated with each IPPF Member Association.

### 2.3 Study Population

From July through December 2023, we recruited participants using a multi-pronged convenience sampling strategy across both healthcare and community settings. In clinic-based settings (e.g., clinics, pharmacies, and health registries), reproductive-age men between the ages of 18 and 50 years old were approached sequentially by trained, non-clinical research staff after receiving services. In parallel, community-based recruitment was conducted via flyers, social media advertisements, and outreach in community gathering spaces, with interested individuals contacting study staff to receive a survey link. This dual strategy was designed to avoid only surveying individuals biased towards healthcare uptake, capture both contraceptive-seeking and non-seeking populations, and improve diversity across sociodemographic and geographic groups. Eligibility was limited to those men who reported being sexually active with a female partner within a year of survey and who denied involvement in a pregnancy, trying to conceive, history or suspicion of infertility, and history of permanent male or female contraception more than 5 years prior to survey. We additionally asked male participants to recruit their female partners for their perspectives, including whether they would trust their male partners to use new MCs. Male partners were informed that they would not have access to the female survey content or responses to preserve independent reporting. Participants were not financially compensated for participation. We aimed to recruit approximately half from each group. Local investigators divided recruitment quotas across several geographic regions to improve sample diversity according to urban versus rural, socioeconomic status, language/dialect, and religion, with 12 recruiting catchments in Colombia, 6 in Ghana, 7 in Morocco, and 6 in Togo.

### 2.4 Survey Design

Attitudes towards new MCs were embedded within a larger survey examining attitudes towards existing contraceptive methods and contraceptive decision-making. The male survey consisted of 77 items, while the female survey consisted of 64 (Appendices 1 and 2). All were multiple choice, except for one open-ended question. The master survey was written in English, professionally translated into the respective main local language (e.g., Spanish in Colombia, French in Togo, Arabic in Morocco), and reviewed and revised by local investigators to ensure all questions and answer choices were appropriately translated for their context. The translated surveys were back translated in line with WHO standards to ensure the translation accurately reflected the original text.

Relevant to the primary objective, men were asked via 4-point Likert scale, “If available and as effective as a female contraceptive pill, would you be willing to try a new method of male contraception to prevent pregnancy?” Respondents had the options of 1 (definitely not) to 4 (definitely yes). Men were additionally asked to select: all applicable reasons for why they might use new MCs from a pre-populated list (e.g., prevent pregnancy, avoiding having to use condoms, have more control, avoid getting trapped, etc.); their preferred formulation or route of administration; dosing schedule; preference over condoms; and preferred location to obtain the drug. Females were asked 4-point Likert items about trusting their male partner’s use of new MCs (“Would you trust your partner to consistently and correctly use a new method of male contraception?) and how it might impact the use of their current method of contraception (“If your male partner was using a new method of male contraception, would you stop using your own method?).

The survey also collected socio-demographics (e.g., age, education, occupation), reproductive histories (e.g., history of pregnancy or pregnancy involvement; experience of an unintended pregnancy, miscarriage, or abortion), experience of reproductive coercion (e.g., “My partner has made it difficult for me or stopped me from preventing a pregnancy or impregnating her when I wanted”), current contraceptive use, contraceptive attitudes, and plans for future pregnancy (e.g., “Are you planning to have a child or another child in the future?”).

To explore the influence of gender-equitable attitudes on willingness to use new MCs, the survey incorporated the Population Council and Instituto Promundo’s Gender Equitable Men (GEM) scale, which has been adapted across cultural settings [19] [20] as a reliable measure of gender-equitable beliefs. In addition, the GEM scale was previously incorporated into an online survey examining willingness to use new MCs among an English-speaking US population, noting the independent association of increasing GEMS scores with men’s willingness [21]. The number of items included in the GEMS varies by relevance to the survey setting; we included 22 of the 24 GEMS items, which covered the following domains: gender roles (e.g., “A woman’s most important role is to take care of her home and family”), relationships (e.g., “A couple should decide together if they want to have children”), masculinity (e.g., “To be a man, you need to be tough”), sexuality (e.g., “Men need sex more than women do”), and violence (e.g., “A woman should tolerate violence in order to keep her family together”). Country-specific modifications to the items and number of GEMS survey items are described in published reports, with acceptable internal consistency noted (Crohnbach’s alpha: 0.75–0.88) [22]. For each GEMS item, the most gender-equitable response was scored as 3, while the least was 1, with the primary measure being the sum of all items and for which the lowest and highest sums could be 22 and 66, respectively.

### 2.5 Data Collection

In healthcare settings, all reproductive-age men were approached sequentially by non-clinically affiliated research staff and informed about the survey. All participants were notified of the voluntary nature of the survey and specifically that declining would not impact their care. All participants provided written informed consent before receiving the survey. The survey was self-administered electronically via the World Health Organization’s (WHO) web-based electronic data capture system, OpenClinica, accessible on tablets, computers, or participants’ own devices. If wanting to complete the survey on-site, participants were guided to private spaces with private handheld tablets or computers. Those wanting to complete the survey at home received a unique, individualized web-based link. Female participants received separate surveys and explicit instructions to complete the survey independently of their partner; to minimize the risk of male participants completing female surveys, surveys were individualized via unique links tied to participant IDs.

For the community-based sample, research staff distributed study flyers in gathering areas for men and posted digital versions to relevant social media channels. Flyers included contact information for research staff who upon contact, provided each interested participant with a unique, web-based survey link. Participants recruited from community centers and gathering places were offered tablet computers for survey completion, or if preferred, received a unique link for completion on their own mobile devices in a location of their choice. The web-based surveys included an informed consent page that required review and approval before proceeding onto the survey. To accommodate varying literacy levels and language proficiency, research staff could provide interviewer-administered entry across both clinic and community samples.

### 2.6 Data Analysis

We present descriptive statistics on men and their female partner’s willingness to use new MCs. As male participation did not depend on successful recruitment of a female partner, male respondents were included regardless of whether their partner completed the survey. Given our primary interest in the attitudes of men at varying levels of relationship commitment, a subset analysis of linked male and female partner data with interest in discordant attitudes and associated factors will be examined in future manuscripts. To explore the role of sociodemographic factors and reproductive experiences and goals on willingness to use new MCs, we collapsed the 4-point Likert items into binary willingness and unwillingness to use new MCs. We conducted bivariate analyses (e.g., X2, t-tests) of new MC willingness against sociodemographic and reproductive factors, the significant results of which, at the p<0.1 level, informed logistic regression modeling for predictive factors. Logistic regression models incorporated significantly associated characteristics from the bivariate analyses, as well as conceptually relevant variables (e.g. age). To improve model stability and reduce collinearity, we collapsed categorical variables with small cell sizes and concern for overlapping constructs (variable inflation factors > 5), including relationship status (e.g., never married/separated/divorced vs not married vs cohabiting) and religious affiliation (any religion vs none), respectively. No variables retained in the final model demonstrated problematic collinearity. Regressions were conducted for the complete sample, as well as by gender, using the statistical package STATA/SE (version 14.2).

## 3 Results

We received 2123 survey entries (response rate: 84.1%), 91.6% of which were complete (N=1945; 22.6% Colombia; 25.2% Ghana; 26.8% Morocco; 25.3% Togo). Approximately half of the surveys came from healthcare versus community settings (51.5% vs. 48.5%). Our sample included a diverse group of men (n=994) and their female partners (n=951): mean age 30.8 years (SD 7.6). More than half (64.0%, n=1244) of the men and women surveyed reported no more than a secondary school education. Half (50.6%, n=985) identified as Christian/Catholic and 38.1% (n=741) identified as Muslim. With respect to occupation, 21.2% (n=412) were in professional/technical professions, 23.7% (n=461) in manual labor, 13.0% (n=253) were unemployed or not working, and 12.3% (n=239) were in agriculture (See Table 1).

In terms of relationship and reproductive background, more than half were married (54.7%, n=1063); 15.2% reported currently having more than one sex partner (23.8% men versus 6.4% female respondents). Nearly three-quarters (71.5%, n=1390) reported ever being pregnant or getting someone pregnant; 23.1% (n=450) reported an unintended pregnancy. More than half of men and their female partners (58.7%, n=1143) similarly reported wanting to father or bear a child in the future. Reproductive coercion within the relationship was reported by 13.7% (n=267), which while significantly varying by country, was not by gender (13.4% men vs. 14.1% female partner, p=0.65). Our population’s mean GEMS score was 52.8±8.0, with the lowest mean in Togo and the highest in Colombia (range: 47.8 to 60.7; See Table 2).

For contraceptive use: 13.7% (n=260) reported not using any contraception; 11.4% (n=216) reported using the most effective methods (e.g., implants or intrauterine devices); 33.8% (n=639) reported using medium effectiveness methods (e.g., pills, patches, rings, injectables); 41.1% (n=778) used lesser effective methods (e.g., condoms, withdrawal). Men more frequently reported using no or lesser effective methods (p<0.01). Of those using a contraceptive method, 17.1% reported being dissatisfied or very dissatisfied with their method. About a quarter of contraceptive users reported they might not continue their method (26%; see Table 3).

**Figure 1:**
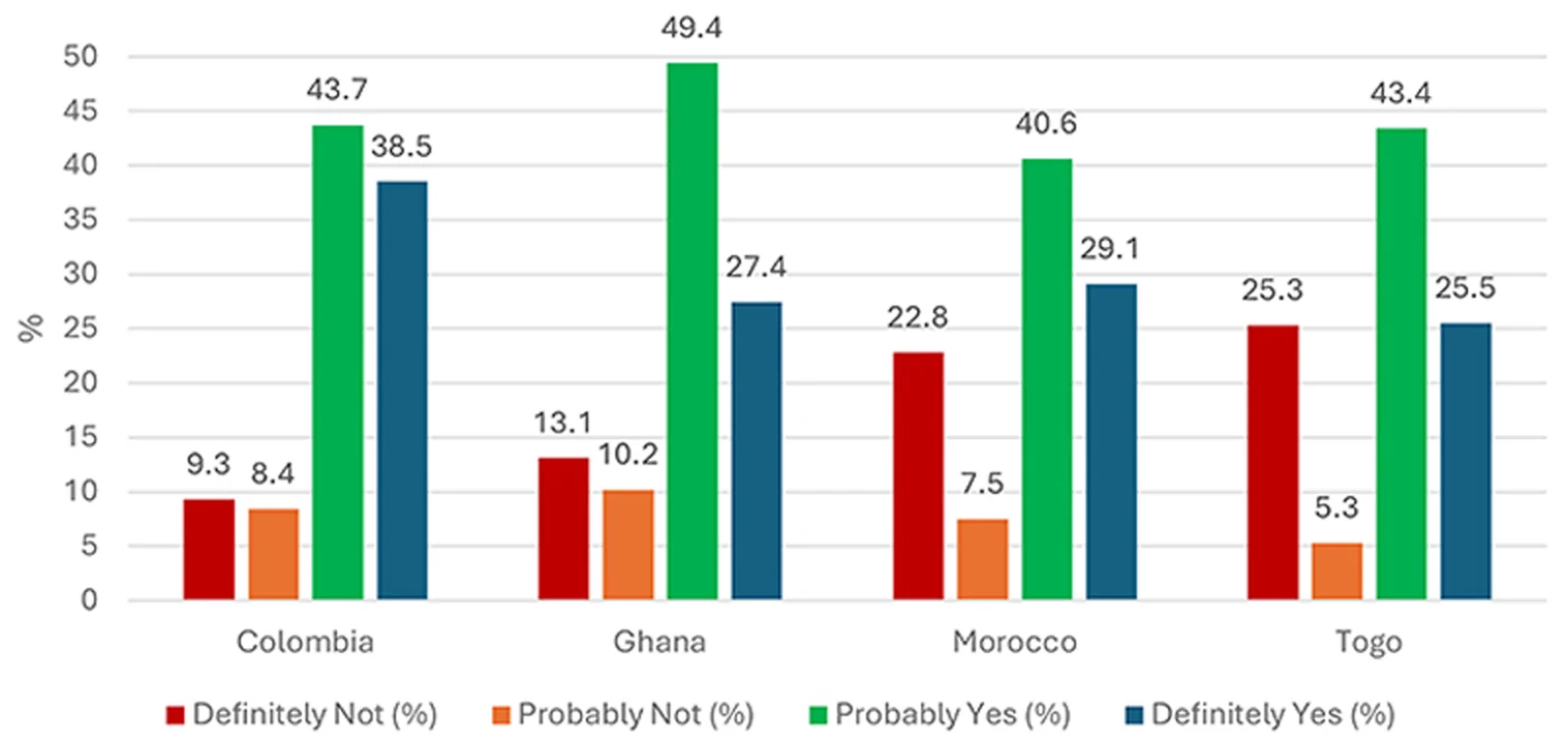
Willingness to use new male contraceptives by country, 2023 (N=1945 men and female partners, p=0.000)

**Figure 2:**
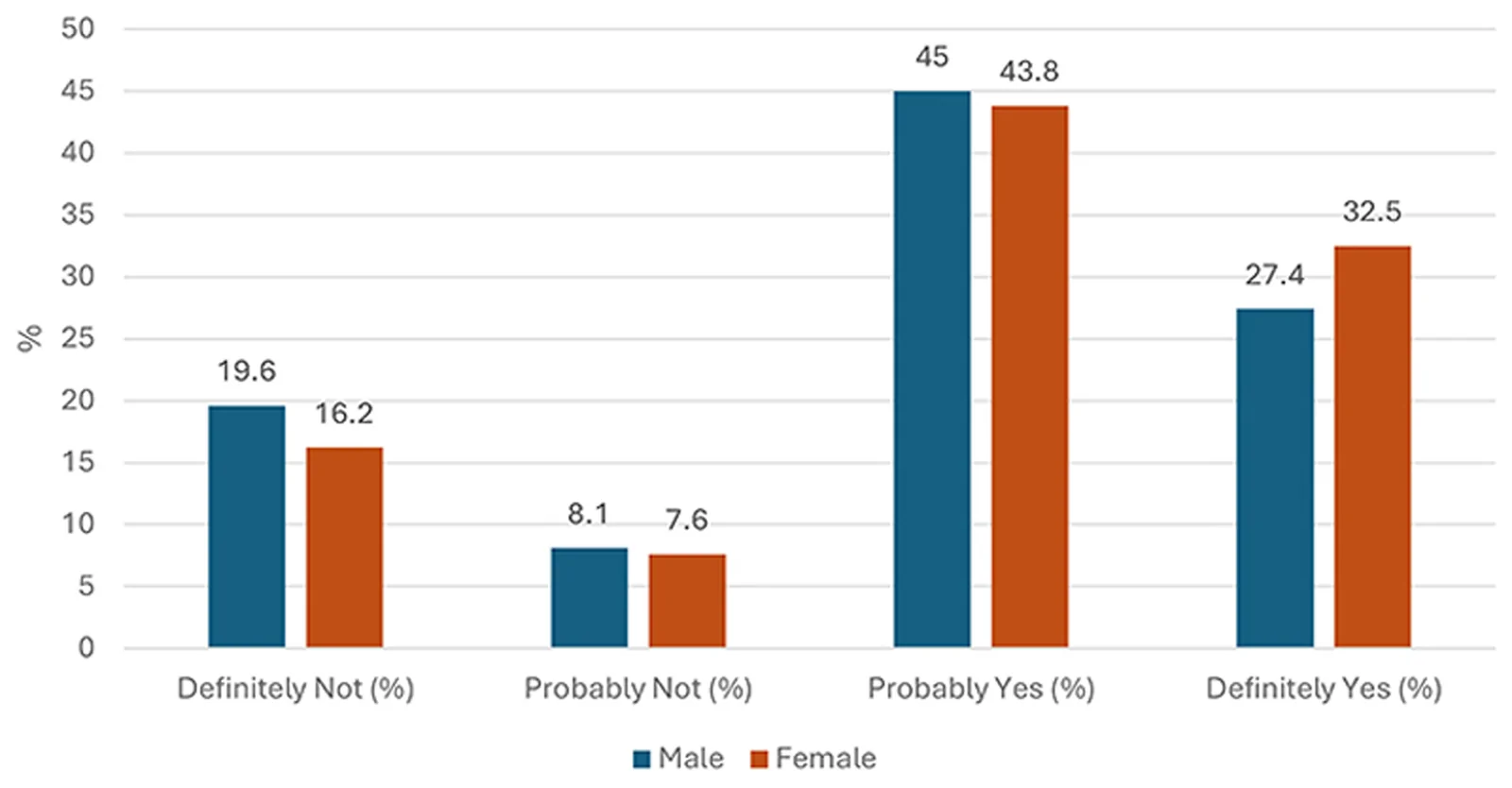
Willingness to use new male contraceptives by sex, 2023 (N=1945 men and female partners, p=0.051)

**Figure 3:**
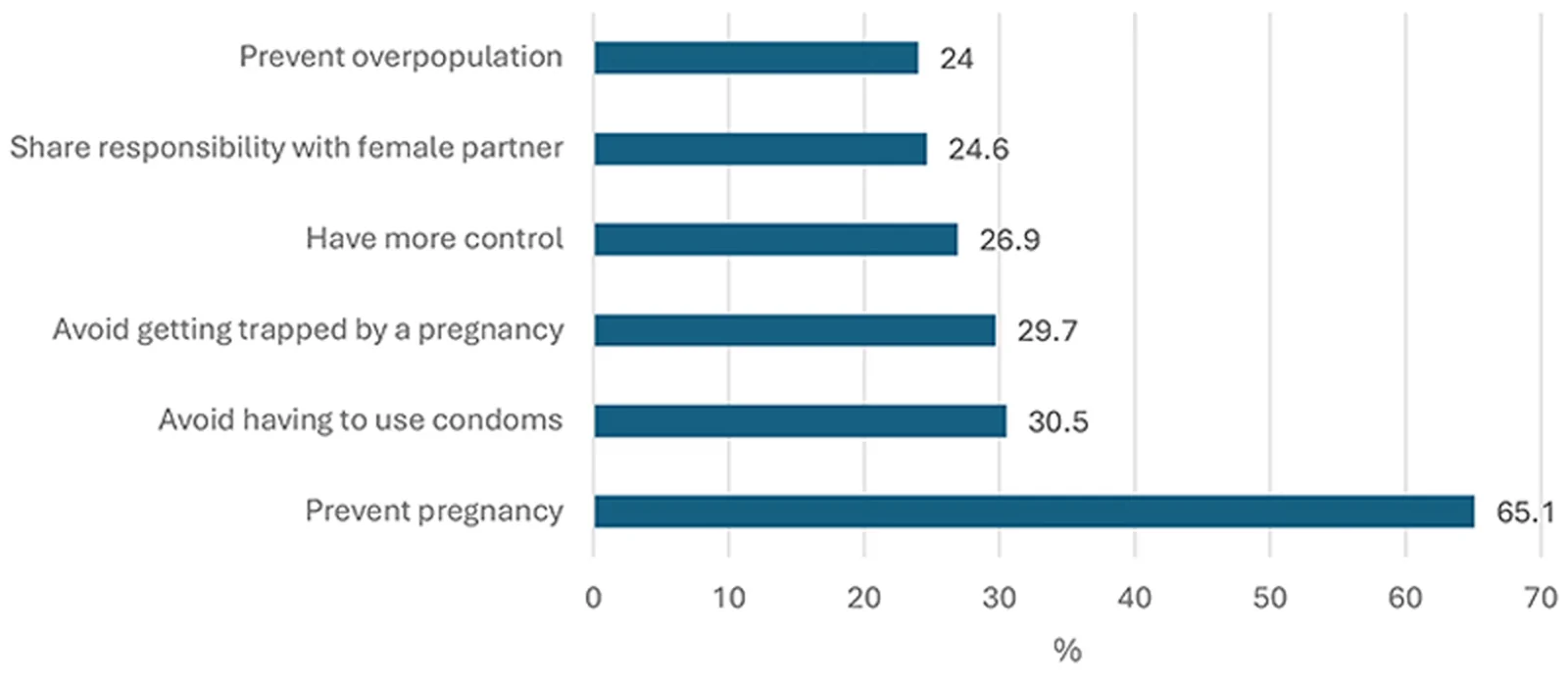
Reasons why men would choose to use a new MC, 2023 (N=994 men)

**Figure 4:**
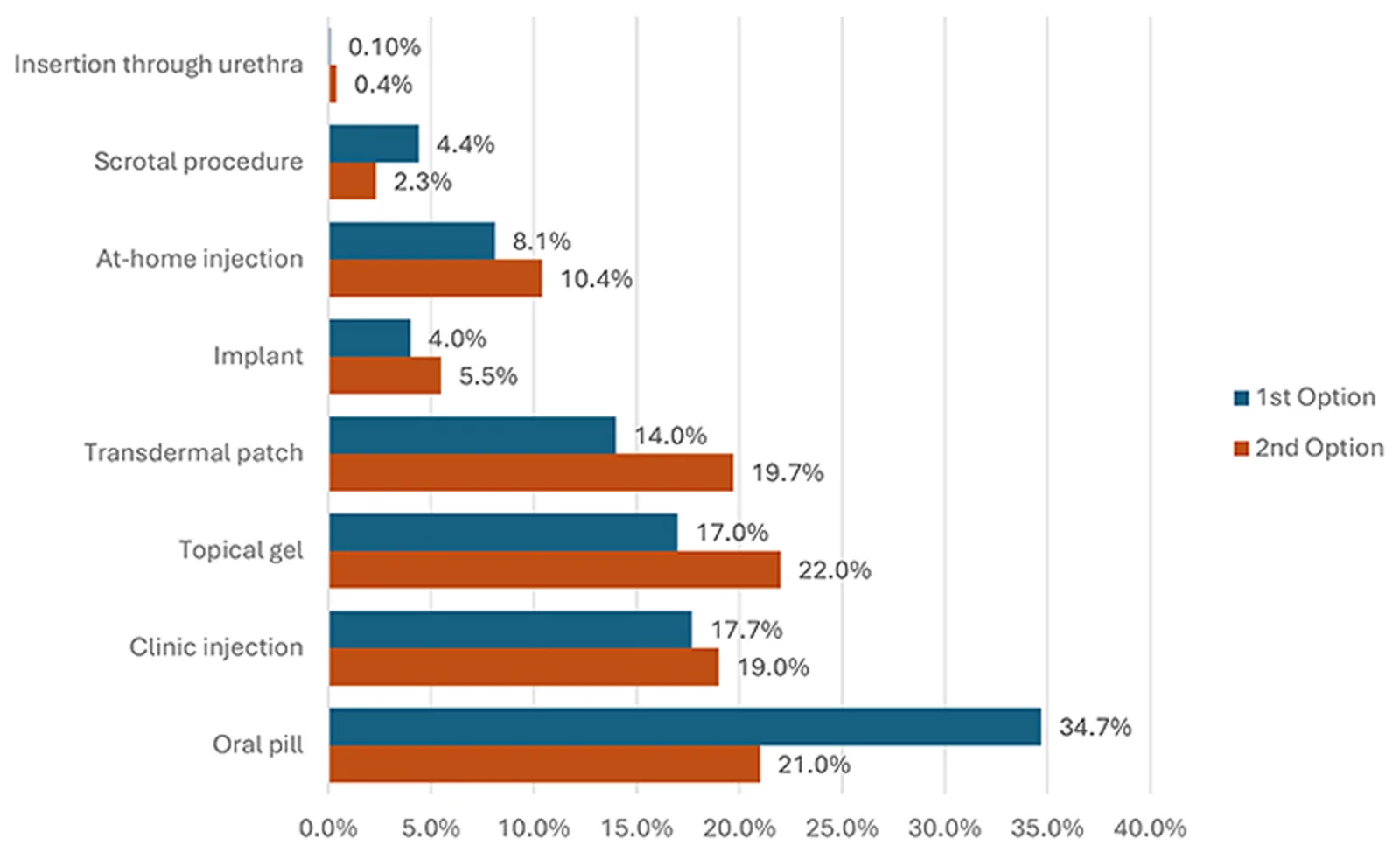
Preferred route of administration among men willing to use a new male contraceptive, 2023 (n=799 men)

**Figure 5:**
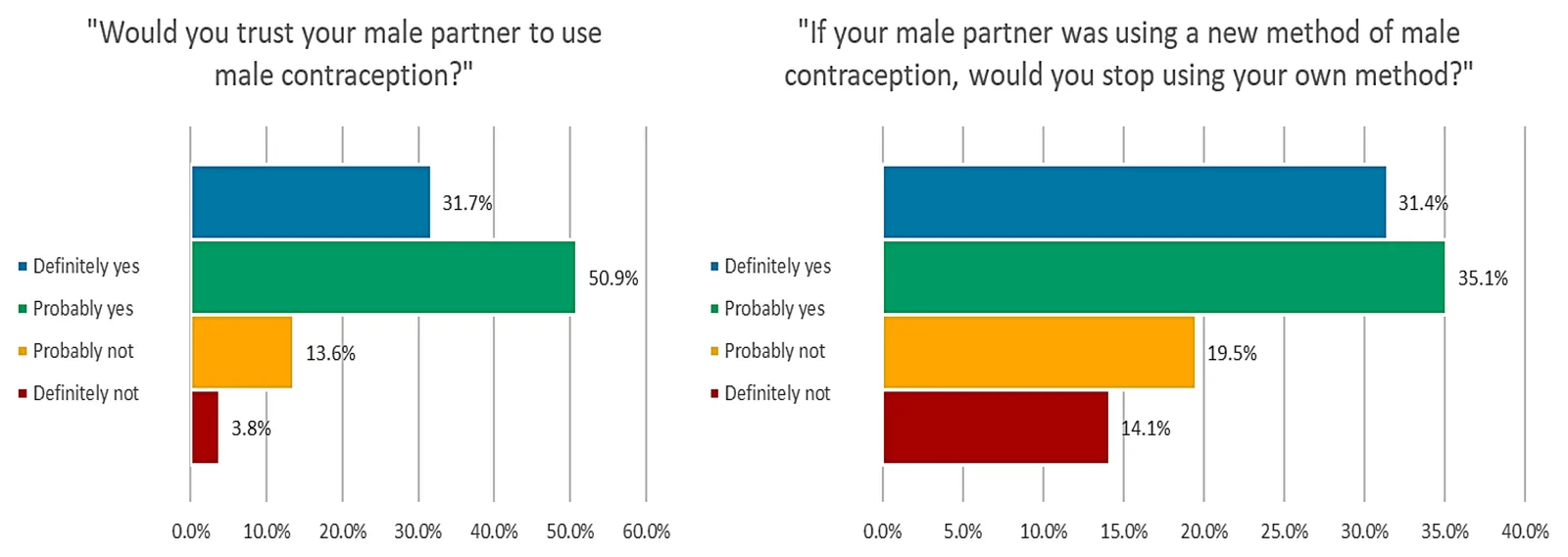
Female partner attitudes towards male partner use of new male contraceptive methods, (n=797 female part

Nearly three-quarters (74.2%, n=1444) of respondents reported they would probably (44.4%) or definitely (29.9%) be willing to use new MCs, ranging from 69.4% at lowest in Togo to highest in Colombia at 82.2% (See Figure 1, p=0.000). Men more commonly reported that they would probably not (8.1% vs. 7.6%) and definitely not (19.6% vs. 16.2%) use new MCs than their female partners, though these differences were not significant (See Figure 2, p=0.051). Male respondents most frequently selected pregnancy prevention (65.1%), being able to avoid using condoms (30.5%), avoid getting trapped by a pregnancy (29.7%), have more control (26.9%), share responsibility with their female partner (24.6%), and prevent overpopulation (24%) as reasons to use new MCs (Figure 3). The most preferred MC route of administration (Figure 4) was oral pills, followed by topical gels and in-clinic injections; 69% reported that their selected new MC formulation would be preferred over condoms. Respondents disproportionately preferred clinics (34.7%), pharmacies (27.2%), and hospitals (26.2%) for obtaining new MCs, as compared to community health workers (9.0%) and mobile clinics (2.5%). More than 80% of female partners reported that they would probably (50.9%) or definitely trust (31.7%) their male partner to use new MCs; more than half would probably (35.1%) or definitely stop (31.7%) using their own method if their male partner used a new MC (Figure 5).

Bivariate analyses examining socio-demographic and reproductive factors linked to men and their female partner’s willingness to use new MCs are displayed in Table 4, with significant associations identified. Our multivariable logistic regression revealed the following factors independently linked to willingness to use new MCs from highest to lowest adjusted odds: seeking or currently using contraception (adjOR 2.44, 95%CI 1.80-3.33), having multiple current sexual partners (adjOR 2.29, 95%CI 1.59-3.31), wanting to avoid a pregnancy at all costs (adjOR 1.88, 95%CI 1.38-2.57), having experienced an unintended pregnancy (adjOR 1.84, 95%CI 1.31-2.58), being married (adjOR 1.69, 95%CI 1.09-2.64), identifying as female (adjOR 1.41, 95%CI 1.08-1.85), and an increasing mean GEMS score (adjOR 1.07, 95%CI 1.05-1.09). Factors decreasing reported willingness to use included: any religious identification (adjOR 0.35, 95%CI 0.14-0.86), and the use of any low (adjOR 0.64, 95%CI 0.44-0.95) or high effectiveness (adjOR 0.49, 95%CI 0.30-0.81) tier contraceptives versus no contraception at all.

In the regression by gender, we noted among men the enduring positive influence of multiple sexual partners (adjOR 2.30, 95%CI 1.52-3.49), history of unintended pregnancy (adjOR 2.11, 95%CI 1.33-3.34), and current use of or desire for contraception (adjOR 1.56, 95%CI 1.02-2.39). Separation by gender additionally revealed decreased odds of willingness to use new MCs among men in Morocco (adjOR 0.50, 95%CI: 0.26-0.95). Among female partners, we noted the positive influence of wanting to avoid a pregnancy (adjOR 2.26-2.27, 95%CI 1.39-3.70), and current use of or desire for contraception (adjOR 4.90, 95%CI 3.03-7.92). In contrast, we also note the negative influence of current use of a low effectiveness (adjOR 0.52, 95%CI 0.28-0.98) or highly effective method (adjOR 0.26, 95%CI 0.12-0.55) among female partners. The total mean GEMS score remained significantly linked to willingness to use new MCs in both men (adjOR 1.06, 95%CI 1.04-1.09) and female partners (adjOR 1.09, 95%CI 1.06-1.13).

## 4 Conclusions

That male partners are commonly implicated in controlling women’s contraceptive access and decision-making [23] raises concerns that new male MCs would be met with resistance when brought to market. However, the results of our multinational survey of men and their female partners in Colombia, Ghana, Togo, and Morocco support the findings of previous population surveys that a substantial proportion of men and their female partners would be willing to use new MCs [12]. Willingness to use new MCs varied across countries from 69.4% in Togo to 82.2% in Colombia, which likely reflects differences in gender norms, contraceptive method availability, and sociocultural context, though these assumptions should be interpreted as exploratory. Importantly, more than 80% of female partners would probably or definitely trust their male partner and 62% would probably or definitely stop using their own method if relying on new MCs.

Our findings on men’s preferences for new MC attributes are consistent with those from the most recent multi-national survey of new MCs, which noted men’s preferences for an oral pill or a topical gel formulations, as well as their preference for new MCs over condoms. However, while *Kaur et al* focused on the influence of product characteristics on men’s willingness to use them, our survey added motivations for using new MCs, as well as the influence of reproductive experiences and attitudes in a comprehensive regression model. When including these factors in the regression, sociodemographic characteristics such as age, education, and occupation were no longer significantly linked; however, having multiple current sex partners and previous involvement in an unintended pregnancy nearly doubled the odds of willingness to use new MCs. We found nearly 25% of men reporting at least one other current sexual partner and approximately 30% reporting wanting to avoid getting trapped by a pregnancy, making it one of the strongest predictors of willingness to use new MCs. These findings question whether willingness to use new MCs represents support of their female partner versus a more self-interested act. While sharing contraceptive responsibility was noted as a motivation to use new MCs among almost 25% of respondents, it was one of the least selected options. As marriage was only a significant predictor of willingness to use new MCs when male and female partner data were analyzed together, the disappearance of this relationship when analyzed by gender suggests that new MCs might offer an opportunity for men to act independently to avoid an unwanted pregnancy, particularly with a secondary partner, rather than an opportunity for shared responsibility. The strength of this relationship may vary by country and its cultural acceptability. However, these hypotheses should be further investigated in future studies via qualitative interviews.

In contrast to *Kaur et al’s r*eport where countries notable for gender inequity had the highest willingness to use new MCs, such as Nigeria (76%) and Bangladesh (76%) versus 49% in the United States, we noted the lowest proportions in similarly disparate countries. According to the World Population Review in 2025, Morocco is among the 10 countries with the least gender equality [15], and 69.4% reported willingness from Morocco versus 82.2% in Colombia, which mirrors the United States’ gender equality index. Correspondingly, men in Morocco reported half the odds of willingness to use new MCs as those from Colombia. Sampling biases may explain these contrasting findings, which our study attempted to minimize by recruiting men from a combination of clinic and community-based settings. Nevertheless, our inclusion of the GEMS score in the survey and its emergence as an independent and significant contributor to willingness to use new MCs for both men and their partners better support the notion of MCs as shared contraceptive responsibility. Gender-equitable attitudes may increase willingness to use male contraception by reframing pregnancy prevention as a shared responsibility and challenging norms that position contraception as primarily a woman’s domain. Taken together with the independent association of involvement in an unplanned pregnancy, these results support previous studies suggesting the potential positive impact of introducing new MCs to men at the time of their partner’s abortion [24].

Our survey also examined current contraceptive use and satisfaction, assuming that individuals on less effective or less satisfactory methods might more readily want to switch to a new MC. However, these associations were not statistically significant in our analysis, suggesting that while new MCs were widely acceptable, uptake may not be dependent on satisfaction with currently used methods. MCs might be expected to be among the wide range of available options that individuals switch between using across their life course.

Until a new MC arrives on the market, estimates of men’s willingness to use them may be aspirational, with men having little lived experience of needing to consistently and correctly use a drug to prevent pregnancy, as well as manage any side effects. As our survey did not include explicit information on potential side effects, nor query participants’ expectations regarding side effects and their severity, our estimates may reflect idealized or hypothetical acceptability. Nevertheless, we contextualize this limitation with findings from *Kaur et al’s* discrete choice experiment noting that side effects were less critical decision-making factors as compared to form and efficacy [14]. Willingness to use may not equate to long-term use, which further limits projections on the potential impact of new MCs.

This study utilized a convenience sampling approach across clinical and community settings and was not designed to produce nationally representative estimates. When considered alongside national demographic estimates from Demographic and Health Surveys (DHS) in Colombia, Ghana, Morocco, and Togo [25], our sample overrepresents individuals with greater access to education, healthcare services, and mobile or digital platforms, and may underrepresent those with lower literacy or more limited access to care. While interviewer-assisted administration was available for participants with limited literacy or language proficiency, particularly in Ghana where the survey was not translated into local languages (e.g., Twi, Ga), participation may have been skewed towards those more comfortable with English. Individuals who chose to participate may have been more comfortable discussing reproductive health or more engaged with contraceptive decision-making, particularly given that recruitment materials mentioned new MCs. In addition, the inclusion of female partners through male participant referral may have resulted in a sample of couples with greater communication or relationship stability than the general population. Our findings should thus be interpreted in the context of a diverse but non-representative sample.

Our findings are also limited by the cross-sectional nature of the survey, for which causal relationships cannot be drawn. Factors linked to willingness to use new MCs in this study are hypothesis-generating and warrant further characterization in future qualitative studies. Further, our survey sites, while selected to offer diversity, may not be generalizable to other settings, including in Asian countries with expected variations in sociocultural norms surrounding contraceptive use.

Nevertheless, these data offer a contrast to stereotypes about men being disinterested about pregnancy prevention and women not wanting to rely on male partners to prevent an unwanted pregnancy. Our findings emphasize the influence of reproductive experiences and attitudes and support the continued development of new MCs for low- and middle-income markets.

## Supplementary Material

Supplementary material

Supplementary material 1

## Figures and Tables

**Table 1:** Socio-demographic characteristics of participants by country, 2023 (N=1945 men and female partners)

	Colombia n=439 (22.6%)	Ghana n=490 (25.2%)	Morocco n=522 (26.8%)	Togo n=494 (25.3%)	Total N=1945 (100%)	p
**Gender**						0.077
- Male	219 (49.9)	245 (50.0)	253 (48.5)	277 (56.1)	994 (51.1)	
- Female	220 (50.1)	245 (50.0)	269 (51.5)	217 (43.9)	951 (48.9)	
**Age**						0.010
- Mean (SD)	28.0 (6.8)	27.9 (6.5)	34.3 (7.2)	32.4 (7.5)	30.8 (7.6)	
**Education**						0.000
- Less than primary school	6 (1.4)	63 (12.9)	115 (22.0)	144 (29.2)	328 (16.9)	
- Primary school completed	22 (5.0)	55 (11.2)	90 (17.2)	86 (17.4)	253 (13.0)	
- Secondary / high school	180 (41.0)	175 (35.7)	128 (24.5)	180 (36.4)	663 (34.1)	
- College / university	196 (44.7)	173 (35.3)	120 (23.0)	69 (14.0)	558 (28.7)	
- Post-graduate completed	35 (8.0)	24 (4.9)	69 (13.2)	15 (3.0)	143 (7.4)	
**Occupation**						0.000
- Professional	127 (28.9)	144 (29.4)	92 (17.6)	49 (9.9)	412 (21.2)	
- Clerical or sales	87 (19.8)	38 (7.8)	60 (11.5)	38 (7.7)	223 (11.5)	
- Agricultural	10 (2.3)	62 (12.7)	19 (3.6)	148 (30.0)	239 (12.3)	
- Household, domestic	41 (9.3)	15 (3.1)	54 (10.3)	47 (9.5)	157 (8.1)	
- Manual labor	56 (12.8)	102 (20.8)	132 (25.3)	171 (34.6)	461 (23.7)	
- Armed forces	5 (1.1)	4 (0.8)	6 (1.2)	2 (0.4)	17 (0.9)	
- Student	74 (16.9)	81 (16.5)	14 (2.7)	14 (2.8)	183 (9.4)	
- Unemployed	39 (8.9)	44 (9.0)	145 (27.8)	25 (5.1)	253 (13.0)	
**Religion**						0.000
- Catholic	260 (59.2)	53 (10.8)	0 (0.0)	139 (28.1)	452 (23.2)	
- Christian	70 (16.0)	270 (55.1)	0 (0.0)	193 (39.1)	533 (27.4)	
- Muslim	0 (0.0)	156 (21.0)	519 (70.1)	66 (8.9)	741 (38.1)	
- Other	75 (17.1)	8 (1.6)	2 (0.4)	77 (15.6)	162 (8.3)	
- None/Atheist	34 (7.7)	3 (0.6)	1 (0.2)	19 (3.9)	57 (2.9)	
**Citizen of country**						0.000
- Yes	415 (94.5)	480 (98.0)	520 (99.6)	484 (98.0)	1899 (97.6)	
- No	24 (5.5)	10 (2.0)	2 (0.4)	10 (2.0)	46 (2.4)	

**Table 2:** Relationship and reproductive characteristics of participants by country, 2023 (N=1945 men and female partners)

	Colombia n=439 (22.6%)	Ghana n=490 (25.2%)	Morocco n=523 (26.9%)	Togo n=494 (25.4%)	Total N=1946 (100%)	p
**Relationship status**						0.000
- Never married	118 (26.9)	171 (34.9)	44 (8.4)	27 (5.5)	360 (18.5)	
- Married	60 (13.7)	237 (48.4)	447 (85.6)	319 (64.6)	1063 (54.7)	
- Separated/widowed	4 (0.9)	5 (1.0)	9 (1.7)	1 (0.2)	19 (1.0)	
- Cohabiting	257 (58.6)	77 (15.7)	22 (4.2)	147 (29.8)	503 (25.8)	
**Multiple current sex partners**						0.000
- No	387 (88.8)	363 (77/9)	459 (92.5)	395 (80.0)	1604 (84.8)	
- Yes	49 (11.2)	103 (22.1)	37 (7.5)	99 (20.0)	288 (15.2)	
**Ever pregnant or gotten someone pregnant**						0.000
- No	203 (46.2)	195 (39.8)	123 (23.6)	34 (6.9)	555 (28.5)	
- Yes	236 (53.8)	295 (60.2)	399 (76.4)	460 (93.1)	1390 (71.5)	
**Experience of an unintended pregnancy**						0.000
- No	391 (89.1)	393 (80.2)	425 (81.4)	286 (57.9)	1495 (76.9)	
- Yes	48 (10.9)	97 (19.8)	97 (18.6)	208 (42.1)	450 (23.1)	
**Experience of a miscarriage**						0.000
- No	395 (90.0)	426 (85.9)	459 (88.1)	346 (70.0)	1626 (83.6)	
- Yes	44 (10.0)	64 (13.1)	62 (11.9)	148 (30.0)	318 (16.4)	
**Experience of an abortion**						0.000
- No	416 (94.8)	433 (88.4)	472 (90.6)	430 (87.0)	1751 (90.1)	
- Yes	23 (5.2)	57 (11.6)	49 (9.4)	64 (13.0)	193 (9.9)	
**Desire for more children**						0.000
- No	190 (43.3)	54 (11.0)	198 (37.9)	112 (22.7)	554 (28.5)	
- Yes	172 (39.2)	403 (82.2)	214 (41.0)	354 (71.7)	1143 (58.7)	
- Unsure	77 (17.5)	33 (6.7)	110 (21.1)	28 (5.7)	248 (12.8)	
**Reproductive coercion in this relationship**						0.113
- No	381 (86.8)	425 (86.7)	461 (88.3)	411 (83.2)	1678 (86.3)	
- Yes	58 (13.2)	65 (13.3)	61 (11.7)	83 (16.8)	267 (13.7)	
**Gender Equitable** **Men Scale**						0.000
- Total Score Mean (SD)	60.7 (4.3)	51.9 (6.4)	51.7 (7.5)	47.8 (7.4)	52.8 (8.0)	

**Table 3:** Contraceptive attitudes, behaviors, and willingness to use new MCs by country, 2023 (N=1945 men and female partners)

	Colombia n=439 (22.6%)	Ghana n=490 (25.2%)	Morocco n=523 (26.9%)	Togo n=494 (25.4%)	Total N=1946 (100%)	p
**Contraceptive interest**						
- Seeking or already using	278 (63.3)	388 (79.2)	406 (77.8)	387 (78.3)	1459 (75.0)	0.000
- Neither seeking nor using	161 (36.7)	102 (20.8)	116 (22.2)	107 (21.7)	486 (21.7)	
**Current contraceptive use (tiered effectiveness)** *						
- None	117 (28.7)	51 (10.7)	24 (4.6)	68 (13.9)	260 (13.7)	0.000
- High effectiveness tier	69 (16.9)	47 (9.9)	39 (7.5)	61 (12.5)	216 (11.4)	
- Medium effectiveness tier	81 (19.9)	100 (21.0)	309 (59.3)	149 (30.5)	639 (33.8)	
- Low effectiveness tier	141 (34.6)	278 (58.4)	149 (28.6)	210 (43.0)	778 (41.1)	
**Satisfaction with current contraception (if using)**						0.000
- Very dissatisfied	18 (5.6)	8 (1.8)	20 (4.0)	4 (1.0)	50 (3.0)	
- Dissatisfied	42 (13.0)	46 (10.6)	100 (19.8)	49 (11.6)	237 (14.1)	
- Satisfied	180 (55.9)	285 (65.7)	293 (58.1)	240 (56.9)	988 (59.3)	
- Very satisfied	82 (25.5)	95 (21.9)	91 (18.1)	129 (30.6)	397 (23.6)	
**Planning to continue current contraception**						0.000
- Definitely not	39 (12.1)	28 (6.5)	59 (11.7)	10 (2.4)	136 (8.1)	
- Probably not	50 (15.5)	74 (17.1)	122 (24.2)	55 (13.0)	301 (17.9)	
- Probably yes	155 (48.1)	239 (55.1)	234 (46.4)	217 (51.4)	845 (50.2)	
- Definitely yes	78 (24.2)	93 (21.4)	89 (17.7)	140 (33.2)	400 (23.8)	
**“How would you feel if you were, or you got someone pregnant now?”**						0.000
- Want to avoid at all costs	245 (55.8)	194 (39.6)	165 (31.6)	253 (51.2)	857 (44.1)	
- Want to avoid, but would be ok	133 (30.3)	175 (35.7)	151 (28.9)	172 (34.8)	631 (32.4)	
- Would be ok either way or would want a pregnancy now	61 (13.9)	121 (24.7)	206 (39.5)	69 (14.0)	457 (23.5)	
**Willingness to use new MCs**						0.000
- Definitely not	41 (9.3)	64 (13.1)	119 (22.8)	125 (25.3)	349 (17.9)	
- Probably not	37 (8.43)	50 (10.2)	39 (7.5)	26 (5.3)	152 (7.8)	
- Probably yes	192 (43.7)	242 (49.4)	212 (40.6)	217 (43.9)	863 (44.4)	
- Definitely yes	169 (38.5)	134 (27.4)	152 (29.1)	126 (25.5)	581 (29.9)	

* High effectiveness: intrauterine devices, subdermal implants; Medium effectiveness: pills, patches, rings, injections; Low effectiveness: condoms, withdrawal, fertility awareness-based methods

**Table 4:** Socio-behavioral factors linked to willingness to use new male contraceptives, 2023 (N=1945 men and female partners)

`	Willingness to use a new male contraceptive		Total (100%)	p	adjOR (95%CI)^a^
	No (501; 25.8%)	Yes (1444; 74.2%)			
**Site**				0.000	
- Colombia	78 (15.6)	361 (25.0)	439 (22.6)		Ref
- Ghana	114 (22.8)	376 (26.0)	490 (25.2)		1.22 (0.73-2.02)
- Morocco	158 (31.5)	364 (25.2)	522 (26.8)		0.83 (0.44-1.53)
- Togo	151 (30.1)	343 (23.8)	494 (25.4)		0.89 (0.55-1.43)
**Gender**				0.049	
- Male	275 (54.9)	719 (49.8)	994 (51.1)		Ref
- Female	226 (45.1)	725 (50.2)	951 (48.9)		1.34 (1.03-1.74)
**Age**				0.230	
- Mean (SD)	31.1 (7.5)	30.7 (7.6)	30.8 (7.6)		--
**Education**				0.000	
- Less than primary school	123 (24.6)	205 (14.2)	328 (16.9)		Ref
- Primary school complete	83 (16.6)	170 (11.8)	253 (13.0)		0.99 (0.66-1.47)
- Secondary / high school	155 (30.9)	508 (35.2)	664 (34.1)		1.17 (0.81-1.68)
- College / university	110 (22.0)	448 (31.0)	558 (28.7)		1.32 (0.85-2.05)
- Post-graduate complete	30 (6.0)	113 (7.8)	143 (7.4)		1.29 (0.71-2.36)
**Occupation**				0.000	
- Professional	93 (18.6)	319 (22.1)	412 (21.2)		Ref
- Clerical or sales	39 (7.8)	184 (12.7)	223 (11.5)		1.58 (0.99-2.53)
- Agricultural	88 (17.6)	151 (10.5)	239 (12.3)		1.18 (0.72-1.94)
- Household, domestic	42 (8.4)	115 (8.0)	157 (8.1)		1.11 (0.65-1.92)
- Manual labor	126 (27.1)	325 (22.5)	461 (23.7)		1.30 (0.86-1.98)
- Armed forces	2 (0.4)	15 (1.0)	17 (0.9)		2.19 (1.13-4.27)
- Student	28 (5.6)	155 (10.7)	183 (9.4)		1.28 (0.82-2.00)
- Unemployed	73 (14.6)	180 (71.2)	253 (13.0)		1.80 (0.74-4.39)
**Religion**				0.004	
- Catholic	94 (18.8)	358 (24.8)	452 (23.2)		0.36 (0.14-0.90)
- Christian	132 (26.4)	401 (27.8)	533 (27.4)		0.33 (0.13-0.82)
- Muslim	227 (45.3)	514 (35.6)	741 (38.1)		0.30 (0.12-0.79)
- Other	40 (8.0)	122 (8.5)	162 (8.3)		0.34 (0.13-0.90)
- None/Atheist	8 (1.6)	49 (3.4)	57 (2.9)		Ref
**Citizen**				0.331	
- Yes	492 (98.2)	1407 (97.4)	1899 (97.6)		--
- No	9 (1.8)	37 (2.6)	46 (2.4)		--
**Relationship status**				0.008	
- Never married	73 (14.6)	287 (19.9)	360 (18.5)		Ref
- Married	292 (58.3)	771 (53.4)	1064 (54.7)		2.05(1.29-3.25)
- Separated or widowed	1 (0.2)	18 (1.3)	19 (1.0)		5.60 (0.69-45.4)
- Cohabiting, unmarried	135 (27.0)	368 (25.5)	503 (25.9)		1.17 (0.76-1.80)
**Multiple current sex partners**				0.001	
- No	444 (89.3)	1160 (83.2)	1605 (84.8)		Ref
- Yes	53 (10.7)	235 (16.9)	288 (15.2)		2.29 (1.58-3.31)
**Ever pregnant or gotten someone pregnant**				0.004	
- No	118 (23.6)	437 (30.3)	555 (28.5)		--
- Yes	383 (76.5)	1007 (69.7)	1390 (71.5)		0.87 (0.61-1.24)
**Unintended pregnancy**				0.000	
- No	418 (83.4)	1077 (74.6)	1495 (76.9)		Ref
- Yes	83 (16.6)	367 (25.4)	450 (23.1)		1.81 (1.28-2.54)
**Miscarriage experience**				0.487	
- No	424 (84.6)	1202 (83.3)	1626 (83.6)		--
- Yes	77 (15.4)	265 (16.7)	318 (16.4)		--
**Abortion experience**				0.004	
- No	466 (93.0)	1285 (89.1)	1751 (90.1)		Ref
- Yes	35 (7.0)	158 (10.9)	193 (9.9)		1.09 (0.68-1.76)
**Desire for more children**				0.002	
- Yes	325 (64.9)	818 (56.7)	1143 (58.7)		Ref
- No	114 (22.8)	440 (30.5)	554 (28.5)		1.11 (0.82-1.50)
- Not Sure	62 (12.4)	186 (12.9)	248 (12.8)		1.13 (0.78-1.63)
**How would you feel if you were, or you got someone pregnant now**				0.000	
- Want to avoid at all costs	169 (33.7)	688 (47.7)	857 (44.1)		1.87 (1.37-2.55)
- Want to avoid, but would be ok	152 (30.3)	479 (33.2)	631 (32.4)		1.68 (1.24-2.27)
- Would be ok either way or would want a pregnancy now	180 (35.9)	277 (19.2)	457 (23.5)		Ref
**Reproductive coercion in this relationship**				0.627	
- No	429 (85.6)	1249 (86.5)	1678 (86.3)		--
- Yes	72 (14.4)	195 (13.5)	267 (13.7)		--
**Gender Equitable Men Scale**					
- Total Score Mean (SD)	49.6 (8.1)	53.9 (7.7)	52.8 (8.0)	0.000	1.07 (1.05-1.09)
**Contraceptive interest**				0.000	
- Seeking or already using	319 (63.7)	1140 (79.0)	1459 (75.0)		2.44 (1.79-3.32)
- Neither seeking nor using	182 (36.3)	304 (21.1)	486 (25.0)		Ref
**Current contraceptive use (tiered effectiveness)**				0.058	
- None	86 (17.3)	174 (12.5)	260 (13.7)		Ref
- High effectiveness tier	55 (11.0)	161 (11.5)	216 (11.4)		0.49 (0.30-0.81)
- Medium effectiveness tier	156 (31.3)	483 (34.6)	639 (33.8)		0.86 (0.56-1.33)
- Low effectiveness tier	201 (40.4)	577 (41.4)	778 (41.1)		0.63 (0.43-0.94)
**Satisfaction with current contraception (if using)**				0.104	
- Dissatisfied or very dissatisfied	60 (14.5)	227 (17.9)	287 (17.1)		--
- Satisfied or very satisfied	355 (85.5)	1040 (82.1)	1395 (82.9)		--
**Planning to continue current contraception**				0.116	
- Definitely or probably not	120 (28.9)	317 (25.0)	437 (26.0)		--
- Definitely or probably yes	295 (71.1)	950 (75.0)	1245 (74.0)		--
					

a variables included in the logistic regression model at the p<0.1 level

**Table 5:** Socio-behavioral factors linked to willingness to use new male contraceptives by gender, 2023 (N=994 men and 951 female partners)

	Male partner adjOR (95%CI)	Female partner adjOR (95%CI)
**Site**		
- Colombia	Ref	Ref
- Ghana	0.94 (0.47-1.88)	1.58 (0.71-3.54)
- Morocco	0.75 (0.32-1.73)	0.92 (0.34-2.47)
- Togo	0.86 (0.46-1.60)	0.92 (0.41-2.07)
**Education**		
- Less than primary school	Ref	Ref
- Primary school complete	0.97 (0.54-1.72)	0.85 (0.47-1.54)
- Secondary / high school	1.20 (0.70-2.04)	1.09 (0.63-1.88)
- College / university	1.69 (0.90-3.16)	0.85 (0.43-1.67)
- Post-graduate complete	1.72 (0.75-3.92)	0.72 (0.27-1.89)
**Occupation**		
- Professional	Ref	Ref
- Clerical or sales	1.45 (0.80-2.60)	1.76 (0.78-3.96)
- Agricultural	1.23 (0.65-2.34)	1.07 (0.46-2.50)
- Household, domestic	1.55 (0.37-6.47)	0.78 (0.36-1.60)
- Manual labor	1.57 (0.93-2.66)	1.03 (0.48-2.14)
- Armed forces	3.85 (0.80-18.47)	1.76 (0.80-4.29)
- Student	1.44 (0.68-3.02)	0.95 (0.46-1.85)
- Unemployed	1.93 (0.78-4.82)	**
**Religion**		
- Catholic	0.47 (0.15-1.48)	0.23 (0.05-1.15)
- Christian	0.53 (0.16-1.68)	0.17 (0.03-0.87)
- Muslim	0.34 (0.10-1.14)	0.25 (0.05-1.34)
- Other	0.50 (0.15-1.66)	0.17 (0.03-0.95)
- None/Atheist	Ref	Ref
**Relationship status**		
- Never married	Ref	Ref
- Married	2.15 (1.15-4.01)	2.10 (1.00-4.40)
- Separated or widowed	3.79 (0.40-35.72)	**
- Cohabiting, unmarried	1.00 (0.56-1.79)	1.58 (0.78-3.22)
**Multiple current sex partners^a^**	2.27 (1.49-3.44)	1.98 (0.80-4.90)
**Unintended pregnancy^a^**	2.01 (1.27-3.20)	1.60 (0.93-2.77)
**Abortion experience^a^**	1.01 (0.55-1.87)	1.05 (0.47-2.32)
**Desire for more children**		
- Yes	Ref	Ref
- No	1.23 (0.81-1.89)	0.98 (0.62-1.54)
- Not Sure	0.94 (0.58-1.52)	1.59 (0.86-2.94)
**How would you feel if you were, or you got someone pregnant now**		
- Want to avoid at all costs	1.47 (0.96-2.25)	2.38 (1.45-3.89)
- Want to avoid, but would be ok	1.25 (0.82-1.89)	2.22 (1.38-3.56)
- Would be ok either way or would want a pregnancy now	Ref	
**Gender Equitable Men Scale^b^**	1.06 (1.03-1.09)	1.09 (1.06-1.13)
**Contraceptive interest (seeking or already using)**	1.54 (1.00-2.37)	4.96 (3.05-8.07)
**Current contraceptive use (tiered effectiveness)**		
- None	Ref	Ref
- High effectiveness tier	0.77 (0.38-1.57)	0.26 (0.12-0.56)
- Medium effectiveness tier	0.92 (0.50-1.67)	0.70 (0.36-1.37)
- Low effectiveness tier	0.67 (0.40-1.12)	0.56 (0.30-1.07)

a Reference group set as “No”

b Change in odds for each unit increase in total GEMS score

c Reference group set as “Neither seeking nor using contraception”

** Insufficient representation for inclusion in regression

## Implications

Our findings both broaden and deepen published support for new MC development worldwide. Regardless of variations in culture and social norms across populations, our findings emphasize the influence of the individual’s desire to avoid an unwanted pregnancy and gender-equitable attitudes.
