# Supplementary material for: Socio-behavioral factors linked to willingness to use new male contraceptives: a cross-sectional survey of men and female partners across four low- and middle-income countries
Source: Contraception. Author manuscript; Available in PMC 2026 Oct 1. (PMC7619453; doi:10.1016/j.contraception.2026.111507)
Supplement: Supplementary file 2 [file EMS217689-supplement-Supplementary_material_1.pdf]

A66030 - A global study of men and women's male contraceptive knowledge, attitudes, and behaviors (form for men)

CONSENT

International Planned Parenthood Federation

Click here to upload file. (< 100MB)

World Health Organization

Click here to upload file. (< 100MB)

You are being asked to participate in a Global Study on Male Contraception. This research is sponsored by the World Health Organization and seeks to understand men's involvement in family planning and attitudes towards new male contraceptive methods. The survey is voluntary, which means that you do not have to participate. The survey is anonymous and should not take more than 30 minutes to complete.

Have you been informed about the study and do you give your consent to proceed?

◯ 1 - Yes ◯ 2 - No

TRACKING

1. Country:

- ( ) 1 - Colombia
- ( ) 2 - Ghana
- ( ) 3 - Morocco
- ( ) 4 - Thailand
- ( ) 5 - Togo

2. What sex were you assigned at birth?

- ( ) 1 - Male
- ( ) 2 - Female
- ( ) 3 - Other

2s. If other sex, please specify:

....

3. Is your female partner going to participate and complete the survey?

- ( ) 1 - Yes
- ( ) 2 - No

SCREENING

4. How old were you at your last birthday?

....

5. Have you had sex with a female partner in the last 12 months? By "sex," we are referring to any penile-vaginal intercourse.

- ( ) 1 - Yes
- ( ) 2 - No

6. Is your partner currently pregnant or trying to become pregnant?

- ( ) 1 - Yes
- ( ) 2 - No

7. Are you or has someone ever told you or your partner that you are infertile or cannot have a child?

- ( ) 1 - Yes
- ( ) 2 - No

8. Has your partner had a permanent sterilization procedure (vasectomy) performed?

- ( ) 1 - Yes
- (•) 2 - No

8s. Was it more than 5 years ago?

- ( ) 1 - Yes
- ( ) 2 - No

SOCIO-DEMOGRAPHICS

9. What is the highest level of education you completed? *

- 1 - No formal schooling
- 2 - Less than primary school
- 3 - Primary school completed
- 4 - Secondary school / High school (or equivalent) completed
- 5 - College / pre-university / University completed
- 6 - Post-graduate degree completed

10. In a typical month, what is your total household income in GHS (Ghana Cedi), including all wages and grants? *

[0-999,999]

11. What is your occupation? What kind of work do you mainly do? *

- 1 - Professional, technical, managerial
- 2 - Clerical or sales
- 3 - Agricultural (farming, livestock, fishing)
- 4 - Household, domestic, cleaning services
- 5 - Skilled and unskilled manual labor (craftwork, assembly, machine operation)
- 6 - Armed forces

12. What is your religion? *

- 1 - Buddhist
- 2 - Catholic
- 3 - Christian (non-Catholic)
- 4 - Christian (Protestant)
- 5 - Hindu
- 6 - Jewish
- 7 - Muslim
- 8 - Sikh
- 9 - Traditional/folk religion
- 98 - Other, specify:
- 97 - None / atheist
- 99 - Prefer not to answer

12s. If other religion, please specify:

13. Were you born in this country? *

- 1 - Yes
- 2 - No

13s. How long have you lived in this country?

(in years)

REPRODUCTIVE

14. What is your current marital or relationship status? *

- 1 - Never Married
- 2 - Currently Married
- 3 - Separated
- 4 - Divorced
- 5 - Widowed
- 6 - Cohabiting

15. How many different people have you had sex with in the last 12 months? By "sex," we are referring to any penile-vaginal intercourse. *

[Enter 999 if refused to answer]

16. How many times have you impregnated someone in your life? *

16s1. How many of those pregnancies were unintended?

16s2. How many of those ended in a miscarriage or pregnancy loss?

16s3. How many of those ended by an abortion or were terminated early?

17. Are you planning to have a child or another child in the future? *

- 1 - Yes
- 2 - No
- 3 - Not sure

18. Which CONTRACEPTIVE METHOD did you or your partner use most often to prevent a pregnancy? *

- 1 - Female sterilization
- 2 - Male sterilization
- 3 - Intrauterine device
- 4 - Injectables
- 5 - Implants
- 6 - Pill
- 7 - Condom
- 8 - Female condom
- 9 - Emergency contraception
- 10 - Standard days method
- 11 - Lactational amenorrhea
- 12 - Rhythm method
- 13 - Withdrawal
- 96 - Don't know
- 97 - Never used
- 98 - Other
- 99 - Did not have sex in the last month

19. How satisfied were you with this method of contraception? *

- 1 - Very dissatisfed
- 2 - Dissatisfied
- 3 - Satisfied
- 4 - Very satisfied

20. How likely are you to continue using this method of contraception in the future? *

1 - Definitely not | 2 - Probably not | 3 - Probably yes | 4 - Definitely yes

21. Would you be willing to obtain a vasectomy (male sterilization) in the future? *

1 - Definitely not | 2 - Probably not | 3 - Probably yes | 4 - Definitely yes

22. Who decides on what method of contraception you and your partner use? *

1 - Mostly you | 2 - More you | 3 - Shared equally | 4 - More partner | 5 - Mostly partner

23. Who decides when and how many children you and your partner will have? *

1 - Mostly you | 2 - More you | 3 - Shared equally | 4 - More partner | 5 - Mostly partner

24. How DIFFICULT or EASY is it for you TO TRUST YOUR PARTNER to always use contraception? *

1 - Very difficult | 2 - Somewhat difficult | 3 - Somewhat easy | 4 - Very easy | 9 - I am the only one who uses contraception

25. How important is your partner's opinion about what contraception you use? *

1 - Not at all important | 2 - Slightly important | 3 - Somewhat important | 4 - Very important

26. Which statement best describes how you feel about impregnating someone at this time? *

1 - Want to avoid at all costs | 2 - Want to avoid, but would be ok if it happened | 3 - Would be ok either way | 4 - Want a pregnancy at this time

27. How upset would you be if you impregnated someone right now? *

1 - Very upset | 2 - Somewhat upset | 3 - Somewhat pleased | 4 - Very pleased

GENDER EQUITY AND MASCULINITY SCALE (GEMS)

28. A woman's most important role is taking care of her home and family *

○ 1 - Strongly agree ○ 2 - Somewhat agree ○ 3 - Do not agree

29. Changing diapers, giving kids a bath, & feeding kids are a mother's responsibility *

○ 1 - Strongly agree ○ 2 - Somewhat agree ○ 3 - Do not agree

30. A man should have the final word about decisions in his home *

○ 1 - Strongly agree ○ 2 - Somewhat agree ○ 3 - Do not agree

31. A woman should tolerate violence in order to keep her family together *

○ 1 - Strongly agree ○ 2 - Somewhat agree ○ 3 - Do not agree

32. There are times when a woman deserves to be beaten *

○ 1 - Strongly agree ○ 2 - Somewhat agree ○ 3 - Do not agree

33. If someone insults a man, he should defend his reputation, with force if he has to *

○ 1 - Strongly agree ○ 2 - Somewhat agree ○ 3 - Do not agree

34. Men need sex more than women do *

○ 1 - Strongly agree ○ 2 - Somewhat agree ○ 3 - Do not agree

35. It is the man who decides when to have sex and what type of sex to have *

○ 1 - Strongly agree ○ 2 - Somewhat agree ○ 3 - Do not agree

36. Men should be embarrassed if unable to get an erection *

○ 1 - Strongly agree ○ 2 - Somewhat agree ○ 3 - Do not agree

37. A man needs other women, even if things with his wife are fine *

○ 1 - Strongly agree ○ 2 - Somewhat agree ○ 3 - Do not agree

38. To be a man, you need to be tough *

○ 1 - Strongly agree ○ 2 - Somewhat agree ○ 3 - Do not agree

39. It disgusts me when I see a man acting like a woman *

○ 1 - Strongly agree ○ 2 - Somewhat agree ○ 3 - Do not agree

40. I would never have a gay friend *

- ◯ 1 - Strongly agree
- ◯ 2 - Somewhat agree
- ◯ 3 - Do not agree

41. It's important for men to have a male friend to talk about his problems with *

- ◯ 1 - Strongly agree
- ◯ 2 - Somewhat agree
- ◯ 3 - Do not agree

42. It is a woman's responsibility to avoid getting pregnant *

- ◯ 1 - Strongly agree
- ◯ 2 - Somewhat agree
- ◯ 3 - Do not agree

43. In my opinion, a woman can suggest using condoms just like a man can *

- ◯ 1 - Strongly agree
- ◯ 2 - Somewhat agree
- ◯ 3 - Do not agree

44. If a guy gets a woman pregnant, the child is responsibility of both *

- ◯ 1 - Strongly agree
- ◯ 2 - Somewhat agree
- ◯ 3 - Do not agree

45. It is important that a father is present in the lives of his children, even if he is no longer with the mother *

- ◯ 1 - Strongly agree
- ◯ 2 - Somewhat agree
- ◯ 3 - Do not agree

46. A couple should decide together if they want to have children *

- ◯ 1 - Strongly agree
- ◯ 2 - Somewhat agree
- ◯ 3 - Do not agree

47. A man and a woman should decide together what type of contraceptive to use *

- ◯ 1 - Strongly agree
- ◯ 2 - Somewhat agree
- ◯ 3 - Do not agree

48. A real man produces a male child *

- ◯ 1 - Strongly agree
- ◯ 2 - Somewhat agree
- ◯ 3 - Do not agree

49. Only when a woman has a child is she a real woman *

- ◯ 1 - Strongly agree
- ◯ 2 - Somewhat agree
- ◯ 3 - Do not agree

REPRODUCTIVE AUTONOMY SCALE

50. My partner has made it difficult for me or stopped me from preventing a pregnancy or impregnating her when I wanted. *

○ 1 - Strongly disagree ○ 2 - Disagree ○ 3 - Agree ○ 4 - Strongly agree

51. It is easy for me to talk to my partner about sex or preventing pregnancy. *

○ 1 - Strongly disagree ○ 2 - Disagree ○ 3 - Agree ○ 4 - Strongly agree

52. Who has the most say about if/when to have a baby in your life and how to prevent/plan a pregnancy? *

○ 1 - Me ○ 2 - My sexual partner ○ 3 - Both of us

○ 4 - Someone else, such as a parent or mother/father-in-law

MALE CONTRACEPTION

53. In my opinion, condoms are too much trouble. *

1 - Strongly disagree | 2 - Disagree | 3 - Agree | 4 - Strongly agree

54. I don't think condoms interfere with the enjoyment of sex. *

1 - Strongly disagree | 2 - Disagree | 3 - Agree | 4 - Strongly agree

55. I just don't like the idea of using a condom. *

1 - Strongly disagree | 2 - Disagree | 3 - Agree | 4 - Strongly agree

56. Withdrawal or pulling out is an effective method of preventing pregnancy. *

1 - Strongly disagree | 2 - Disagree | 3 - Agree | 4 - Strongly agree

57. A man who has a vasectomy is acting responsibly. *

1 - Strongly disagree | 2 - Disagree | 3 - Agree | 4 - Strongly agree

58. A man having a vasectomy is safer than a woman having a tubal ligation. *

1 - Strongly disagree | 2 - Disagree | 3 - Agree | 4 - Strongly agree

59. Having a vasectomy will negatively impact my sex life. *

1 - Strongly disagree | 2 - Disagree | 3 - Agree | 4 - Strongly agree

60. Having a vasectomy will negatively impact my health in the long-term. *

1 - Strongly disagree | 2 - Disagree | 3 - Agree | 4 - Strongly agree

NEW MALE CONTRACEPTION

61. If available, would you be willing to use a new method of male contraception to prevent pregnancy? *

1 - Definitely not | 2 - Probably not | 3 - Probably yes | 4 - Definitely yes

62. The following are some reasons why men might choose to use a new male contraceptive. Please select all that apply to you. *

- 1 - Prevent pregnancy
- 2 - Avoid having to use condoms
- 3 - Have more control
- 4 - Avoid getting trapped by pregnancy
- 5 - Share responsibility with female partner
- 6 - Avoid side effects from female contraception
- 7 - Prevent overpopulation
- 8 - Other

62s. If other, please provide details:

63. Please review the following forms of new male contraception that are currently in development. Which would be your #1 preference to use? *

- 1 - Oral pill
- 2 - Topical gel on the shoulder
- 3 - Injection given at a clinic
- 4 - Injection given by yourself at home
- 5 - Patch on the skin
- 6 - Implant under the skin
- 7 - Small procedure with cuts in the scrotum in order to occlude or block sperm transport
- 8 - Inserted through the hole where you urinate

64. How much would you prefer this method compared to male condoms? *

1 - Much more | 2 - Somewhat more | 3 - About the same | 4 - Somewhat less | 5 - Much less

65. Please review the following forms of new male contraception that are currently in development. Which would be your #2 preference to use? *

- 1 - Oral pill
- 2 - Topical gel on the shoulder
- 3 - Injection given at a clinic
- 4 - Injection given by yourself at home
- 5 - Patch on the skin
- 6 - Implant under the skin
- 7 - Small procedure with cuts in the scrotum in order to occlude or block sperm transport
- 8 - Inserted through the hole where you urinate

66. How much would you prefer this method compared to male condoms? *

1 - Much more | 2 - Somewhat more | 3 - About the same | 4 - Somewhat less | 5 - Much less

67. How often would you prefer to use/administer/apply the new male contraceptive drug? *

- 1 - Before sex only when needed
- 2 - Once a day
- 3 - Once a week
- 4 - Once a month
- 5 - Once every 3 months
- 6 - Once every 6 months
- 7 - Once a year
- 8 - Once every 3 years
- 9 - Once every 5 years
- 10 - One time procedure only

68. Would you use a new, reversible male contraceptive method if it contained HORMONES? *

1 - Definitely not | 2 - Probably not | 3 - Probably yes | 4 - Definitely yes

69. Would you use a new, reversible male contraceptive method if it required a small SURGERY? *

1 - Definitely not | 2 - Probably not | 3 - Probably yes | 4 - Definitely yes

70. Would you use a new male contraceptive method if there was a risk of MOOD CHANGES that was reversible? *

1 - Definitely not | 2 - Probably not | 3 - Probably yes | 4 - Definitely yes

71. Would you use a new male contraceptive method if there was a risk of FATIGUE that was reversible? *

1 - Definitely not | 2 - Probably not | 3 - Probably yes | 4 - Definitely yes

72. Would you use a new male contraceptive method if there was a risk of LOWER SEX DRIVE that was reversible? *

○ 1 - Definitely not ○ 2 - Probably not ○ 3 - Probably yes ○ 4 - Definitely yes

73. Would you use a new male contraceptive method if there was a small decrease in TESTES size that was reversible? *

○ 1 - Definitely not ○ 2 - Probably not ○ 3 - Probably yes ○ 4 - Definitely yes

74. Understanding that no female contraceptives currently protect against sexually transmitted infections, how important is it that male contraceptives protect against SEXUALLY TRANSMITTED INFECTIONS? *

○ 1 - Very important ○ 2 - Somewhat important ○ 3 - A little important ○ 4 - Not important

75. Please review the following places where a man might go to obtain new male contraceptives. Which would be your #1 preference to go for male contraception? *

○ 1 - Hospital
○ 2 - Health center or clinic
○ 3 - Mobile clinic
○ 4 - Pharmacy/Drug store/Retail store
○ 5 - Village doctor or traditional healer
○ 6 - Community health worker

76. Please review the following places where a man might go to obtain new male contraceptives. Which would be your #2 preference to go for male contraception? *

○ 1 - Hospital
○ 2 - Health center or clinic
○ 3 - Mobile clinic
○ 4 - Pharmacy/Drug store/Retail store
○ 5 - Village doctor or traditional healer
○ 6 - Community health worker

You have reached the end of the survey. You will not be able to make any changes once you close the survey. Please review and revise any answers before clicking the 'Close' button.
